# An Exposed-Core Grapefruit Fibers Based Surface Plasmon Resonance Sensor

**DOI:** 10.3390/s150717106

**Published:** 2015-07-14

**Authors:** Xianchao Yang, Ying Lu, Mintuo Wang, Jianquan Yao

**Affiliations:** College of Precision Instrument and Opto-Electronics Engineering, Key Laboratory of Opto-electronics Information Technology, Ministry of Education, Tianjin University, Tianjin 300072, China; E-Mails: yangxianchao@tju.edu.cn (X.Y.); wo3336903@126.com (M.W.); jqyao@tju.edu.cn (J.Y.)

**Keywords:** exposed-core grapefruit fibers, surface plasmon resonance, *x*- and *y*-polarized, silver layer thickness

## Abstract

To solve the problem of air hole coating and analyte filling in microstructured optical fiber-based surface plasmon resonance (SPR) sensors, we designed an exposed-core grapefruit fiber (EC-GFs)-based SPR sensor. The exposed section of the EC-GF is coated with a SPR, supporting thin silver film, which can sense the analyte in the external environment. The asymmetrically coated fiber can support two separate resonance peaks (*x*- and *y*-polarized peaks) with orthogonal polarizations and *x*-polarized peak, providing a much higher peak loss than *y*-polarized, also the *x*-polarized peak has higher wavelength and amplitude sensitivities. A large analyte refractive index (RI) range from 1.33 to 1.42 is calculated to investigate the sensing performance of the sensor, and an extremely high wavelength sensitivity of 13,500 nm/refractive index unit (RIU) is obtained. The silver layer thickness, which may affect the sensing performance, is also discussed. This work can provide a reference for developing a high sensitivity, real-time, fast-response, and distributed SPR RI sensor.

## 1. Introduction

Propagating at the metal/dielectric interface, surface plasmons are extremely sensitive to changes in the refractive index of the dielectric [1]. Its high sensitivity to the refractive index (RI) variations of the dielectric adjacent to a metal shows great potential in chemical, biomedical, and industrial sensing [2]. Some surface plasmon resonance (SPR) configurations, such as the Kretschmann–Raether prism, can realize extremely high sensitivity, but present a number of disadvantages, such as costly integration, limited mechanical reliability, and difficulties in mass production [3]. Recently, optical fiber-based sensors have become more and more attractive because of their miniaturization, electromagnetic immunity, high sensitivity, and remote sensing capabilities. In most of the fiber-based SPR sensors, cladding is partially or completely stripped from the fiber and the exposed part is coated with a metal layer and then exposed to an analyte [2], which is very difficult and time-consuming. Additionally, the phase matching condition between the core mode and the plasmon mode is not easily achieved, because the fused silica material RI (about 1.45) is usually higher than bordering analyte (about 1.33). Theoretically, phase matching requires equating the propagation constants of the two modes, implying that the effective refractive indices of the two modes have to be similar. The effective RI of a core-guided mode is similar to the background silica, and the effective RI of a plasmonic mode is close to the bordering analyte [4].

The appearance of photonic crystal fibers (PCFs) is a breakthrough in fiber fiber-optic technology, leading to unprecedented properties that overcome many limitations [1]. In contrast with traditional optical fibers, PCFs have several geometric parameters that can be manipulated for larger greater flexibility of design [5]. The existence of air holes provide the possibility to insert analytes, then the mode effective RI could be tunable to manage the anticipated values, solving the phase matching problem. To realize SPR sensing, the air holes are selectively or completely coated with the metal layers, and then filled with analytes. One can also realize the SPR sensing by filling the silver nanowires [6,7]. However, we should note that either coating the metal films or filling analytes and silver nanowires into the air holes is difficult and time-consuming work, which makes it impossible for real-time, fast-response use. Some sensors, based on the D-shaped optical fibers, have been investigated [2,8,9]. The PCF is side-polished to form a flat plane and then coated with a metal film. Compared to the inside coating of the fiber holes, the outside coating is much easier. As the sensing region is exposed to the external environment, the sensors can realize fast-response and real-time sensing. However, the cladding air holes are too small and intensive, and it is very easy to destroy the cladding air holes when polished. Then Because of this, the plane will not be flat and the metal film will not be uniform, which may affect the sensing results. In [9], Tian *et al.* simulated an all-solid photonic fiber with D-shaped structure based surface plasmonic resonance sensor and a sensitivity of 7300 nm/refractive index unit (RIU) can be achieved. Recently, Luan *et al.* also reported a surface plasmon resonance sensor based on D-shaped microstructured optical fiber with hollow core, and identified the sensor sensitivity on wavelength, amplitude, and phase. The wavelength sensitivity they obtained was 2900 nm/RIU when analyte RI changes from 1.33 to 1.34 [2].

In this paper, we design an exposed-core grapefruit fiber (EC-GF)-based SPR sensor to detect analyte RI. Two air hole claddings of the grapefruit fiber are polished and coated with a silver film to form an analyte channel. As the grapefruit fiber air holes are much larger (80 *µ*m) than the ordinary PCF (less than 2 µm), the operation can be much easier than with D-shaped optical fiber sensors, also grapefruit fibers have been produced by many manufacturers, which can be widely and practically used. Unlike the symmetrically coated fibers that support only one single resonance peak, the asymmetrically coated fiber can support two separate peaks, which are *x*-polarized and *y*-polarized peaks, respectively [10]. The *x*-polarized peak provides a much higher peak loss than *y*-polarized, and the *x*-polarized peak has higher wavelength and amplitude sensitivities. A large analyte RI range from 1.33 to 1.42 is calculated by the designed sensor, and an extremely high wavelength sensitivity of 13,500 nm/RIU is obtained. The silver coating’s thickness, which may affect the sensor’s performance, is also discussed. This work can provide a reference for the implementation and application of EC-GF-based SPR sensors or other fibers-based SPR sensing.

## 2. Structure Design and Simulated Modeling

**Figure 1:**
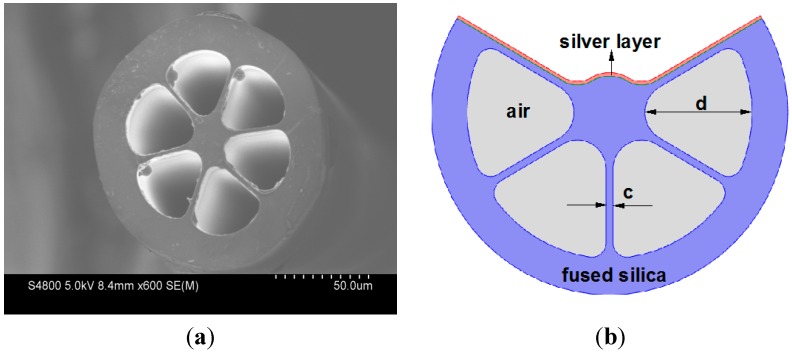
(**a**) Cross-section of the commercial grapefruit fiber; (**b**) Schematic of the designed EC-GF-based SPR sensor.

The cross-section of commercial grapefruit fiber is shown in Figure 1a. The schematic of the designed EC-GF-based SPR sensor is shown in Figure 1b. The thickness of the core struts are c = 2 μm. The diameters of the core and the air holes are d_c_ = 20 μm and d = 80 μm, respectively. The exposed section of the fiber is coated with a 40 nm silver layer. Coating of the metal layers can be performed either with a chemical vapor deposition technique [11] or a wet chemistry deposition technique [12], which is much easier than inside coating of the fiber holes in operation. The refractive index of the EC-GF material is assumed to be 1.45 (fused silica), and the refractive index of the silver is given by the Handbook of Optics [13].

**Figure 2:**
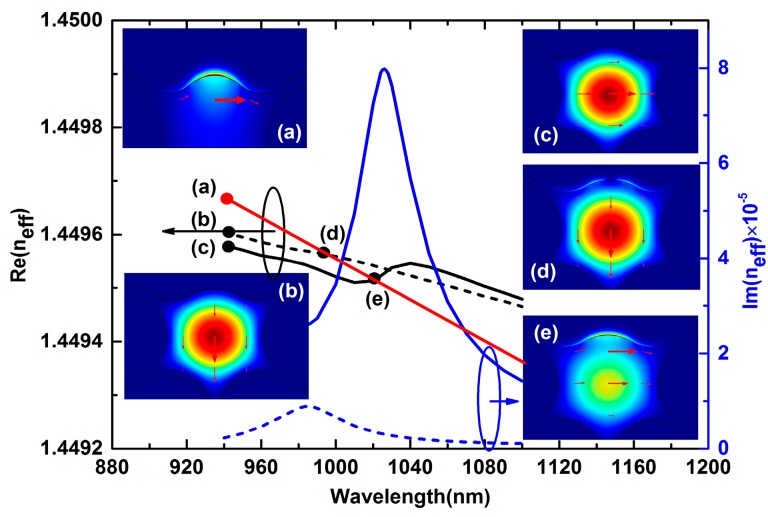
Dispersion relations and electric field distributions of core modes and the plasmon mode with analyte RI n_a_ = 1.42. Insets (**a**) to (**e**) are electric field distributions of plasmon mode and core mode at different wavelengths.

The electromagnetic mode of the sensor is solved by the finite element method (FEM) using COMSOL multiphysics software. Figure 2 shows the dispersion relations and electric field distributions of the core mode and plasmon mode when the RI of the liquid analyte is 1.42. The black solid curve represent the real parts of the effective RI of the *x*-polarized core mode and black dotted curve represent the real parts of the effective RI of *y*-polarized core mode. The red curve represents the plasmon mode. The blue solid curve and blue dotted curve represent the imaginary parts of the effective RI of *x*-polarized core mode and *y*-polarized core mode, respectively. Inset (a) represent electric field distributions of plasmon mode at λ = 1026 nm; (b) represent *y*-polarized core mode at λ = 940 nm; (c) represent *x*-polarized core mode at λ = 940 nm; (d) represent *y*-polarized core mode at λ = 985 nm (phase matching point); and (e) represent *x*-polarized core mode at λ = 1026 nm (phase matching point).

Here, we use the Gaussian-like modes as the core modes, which are best suited for the excitation by standard Gaussian laser sources. As shown in Figure 2, the core modes exhibit strong birefringence, with one mode (*y*-polarized mode see insets (b)) being polarized essentially parallel to the axis of symmetry and the other (*x*-polarized mode see insets (c)) orthogonal to it [2]. Then, there will be two effective RI curves of the core modes, resulting in two intersections (dots (d) and (e)) with the plasmon mode and two resonance peaks for the same analyte index. We can see that the *x*-polarized resonance peak presents a much higher peak loss than *y*-polarized, also the *x*-polarized resonance peak has a higher coupling efficiency (insets (e) and (d)). When the phase matching is satisfied at a certain wavelength, the energy of a core mode is transferred to the plasmon mode and a resonant loss peak will be observed at this wavelength. The variation of the analyte RI will induce changes of the phase matching point between the core mode and the plasmon mode, thus leading to different loss spectra, which can be identified by measuring the peak wavelength shift or transmitted power change.

## 3. Results and Discussion

### 3.1. RI Sensitivity of the Sensor

**Figure 3:**
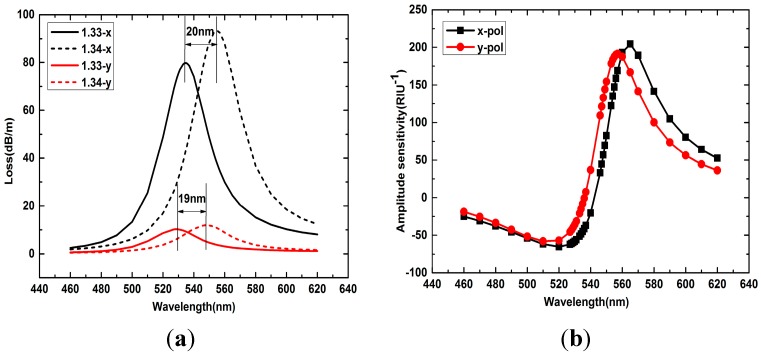
(**a**) Loss spectra of *x*- and *y*-polarized peaks with analyte RI 1.33 and 1.34; (**b**) Amplitude sensitivity of *x*- and *y*-polarized core modes with analyte RI changes from 1.33 to 1.34.

To investigate the sensing performance of the sensor, a large analyte RI range from 1.33 to 1.42 is calculated. Figure 3a shows the loss spectra of the *x*- and *y*-polarized peaks with analyte RI 1.33 and 1.34. The confinement loss is defined as:

(1) $$\alpha_{loss}\left(dB/m\right)=8.686\cdot k_{0}\text{Im}\left[n_{eff}\right]$$

where $k_{0}=2\pi /\lambda$ is the wavenumber with λ in meters and Im(*n_eff_*) is the imaginary part of the mode effective RI. We can see that the *x*-polarized resonance peak presents a much higher peak loss than *y*-polarized, as the *x*-polarized resonance peak has a higher coupling efficiency. When the analyte RI changes from 1.33 to 1.34, both the *x*- and *y*-polarized peaks all shift to the longer wavelength, but the *x*-polarized resonance peak has a larger shift (20 nm) than the *y*-polarized (19 nm). The wavelength sensitivity is defined as:

(2) $$S_{\lambda }(nm/RIU)=\frac{\partial \lambda_{peak}}{\partial n_{a}}$$

where λ_peak_ is the resonance wavelength and n_a_ is the analyte RI. Then the *x*-polarized resonance peak has a higher wavelength sensitivity (2000 nm/RIU) than *y*-polarized (1900 nm/R). If the spectral variation of 1 nm can be accurately detected by the spectrograph, then the sensor’s detection resolution is *R* = 1/*S_λ_*. The resolution of *x*- and *y*-polarized peaks are 5 × 10^−4^ RIU and 5.26 × 10^−4^ RIU, respectively.

Another frequently used detection method is known as the power detection. Assume that the wavelength of the light is λ, and the transmission length is L, then the amplitude sensitivity can be defined as:

(3) $$S\left(RIU^{-1}\right)=\frac{1}{\alpha \left(\lambda ,n_{a}\right)}\frac{\partial \alpha \left(\lambda ,n_{a}\right)}{\partial n_{a}}$$

Figure 3b shows the amplitude sensitivity of the *x*- and *y*-polarized core modes with analyte RI changes from 1.33 to 1.34. From the picture we can see that the *x*-polarized resonance peak has a higher amplitude sensitivity (204 RIU^−1^) than the *y*-polarized (191 RIU^−1^). This is because the *x*-polarized core mode has a larger effective sensing region and stronger mode coupling than the *y*-polarized.

**Figure 4:**
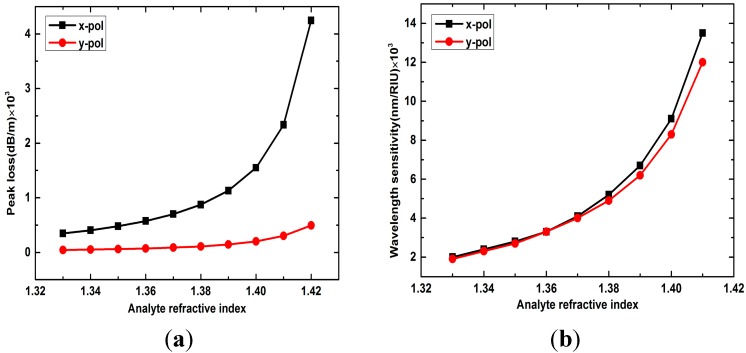
(**a**) *X*- and *y*-polarized peak losses with analyte RI changes from 1.33 to 1.42; (**b**) Wavelength sensitivity of *x*- and *y*-polarized peaks with analyte RI changes from 1.33 to 1.42.

With the analyte RI increasing, as shown in Figure 4a, *x*- and *y*-polarized peak losses all increases, but the *x*-polarized peak loss increases much more quickly than the *y*-polarized, resulting in the gap between the *x*-polarized peak loss and the *y*-polarized getting larger and larger. From Figure 4b we can see that both the *x*- and *y*-polarized peaks show higher sensitivity for the high analyte RI change than the low. For example, the *x*-polarized wavelength sensitivity is 13,500 nm/RIU when analyte RI changes from 1.41 to 1.42, which is much higher than 2000 nm/RIU when analyte RI changes from 1.33 to 1.34. The reason is that when the analyte RI increasing from 1.33 to 1.42, the effective RI of the plasmonic mode is getting more and more close to the effective RI of the core-guided mode (1.45), then the mode coupling will be enhanced, leading to a larger peak loss and higher sensitivity. Considering the wavelength sensitivity and the loss peak amplitude, the *x*-polarized resonance peak is more suitable for RI sensing. The detailed wavelength sensitivities of *x*-polarized peaks when analyte RI increasing from 1.33 to 1.42 is 2000 nm/RIU, 2400 nm/RIU, 2800 nm/RIU, 3300 nm/RIU, 4100 nm/RIU, 5200 nm/RIU, 6700 nm/RIU, 9100 nm/RIU, and 13,500 nm/RIU, respectively. The highest wavelength sensitivity is 13,500 nm/RIU and the minimum resolution is 7.41 × 10^−5^ RIU when the spectrograph resolution is 1 nm, which is higher than other exposed-core fiber based SPR sensors [9,10].

In the fiber fabrication process, we can use higher RI background materials, such as polymer (PMMA n ≈ 1.5) [14], lead silicate (Schott F2, n ≈ 1.62) [15], and bismuth (n ≈ 2.09) [16] to increase the upper detection limit, which can be used to detect some high RI organic chemical liquid analytes like benzene, nitrobenzene and phenylamine [10]. Additionally, we can use lower RI background materials or lower the RI of the core mode by inserting a central air hole [2,4,17] to improve the sensitivity when detecting the low RI liquid analytes.

### 3.2. Influence of Silver Layer Thickness on Sensitivity

**Figure 5:**
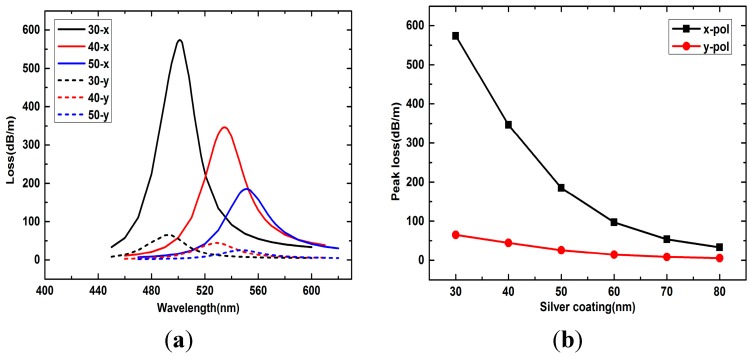
(**a**) Loss spectra of *x*- and *y*-polarized peaks with silver layer thicknesses 30 nm, 40 nm and 50 nm when analyte RI is 1.33; (**b**) *x*- and *y*-polarized peak losses with silver layer thicknesses changes from 30 nm to 80 nm when analyte RI is 1.33.

Surface plasmonic waves are very sensitive to the thickness of the silver layer. As shown in Figure 5a, the loss spectra of the *x*- and *y*-polarized peaks vary considerably with the silver layer thickness changes from 30 nm to 50 nm when analyte RI is 1.33. Generally, *x*- and *y*-polarized peaks all shift to the longer wavelength as the silver layer becomes thicker and the peak losses decrease gradually. As the silver layer thickness continues to increase, from Figure 5b we can see that the decrease of *x*- and *y*-polarized peak losses become slower. Thus, when performing experiments, we can tune the peak loss to a desired value by adjusting the silver layer thickness.

**Figure 6:**
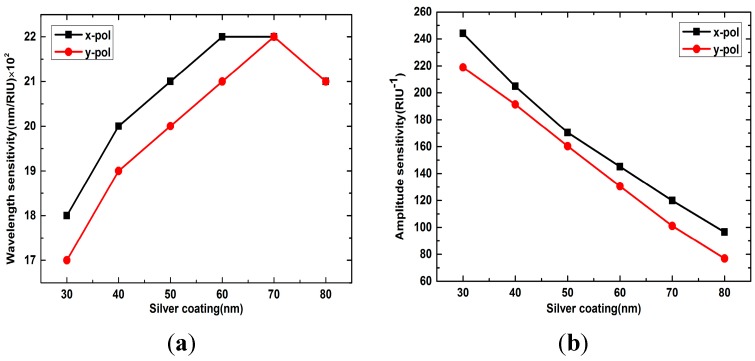
(**a**) Wavelength sensitivity of *x*- and *y*-polarized peaks with silver layer thicknesses change from 30 nm to 80 nm; (**b**) Amplitude sensitivity of *x*- and *y*-polarized peaks with silver layer thicknesses change from 30 nm to 80 nm

Figure 6a shows the wavelength sensitivity of the *x*- and *y*-polarized peaks with different silver layer thicknesses when analyte RI is 1.33. When the silver layer thickness changes from 30 nm to 80 nm, the wavelength sensitivity of *x*- and *y*-polarized peaks all go through a circle of rise and fall, which increase with the increases of silver layer thickness at first. When the silver layer is 70 nm, the sensor has the maximum wavelength sensitivity of 2200 nm/RIU and then it tends to decrease with the continued increase. The *x*-polarized peak always has a higher wavelength sensitivity than the *y*-polarized when the silver layer thickness is below 70 nm, but then they have the same wavelength sensitivity when the thickness is more than 70 nm. Contrary to the wavelength sensitivity, the amplitude sensitivity of the *x*- and *y*-polarized peaks will always reduce with the silver layer thickness increase, as shown in Figure 6b, and the reduction rate is approximately linear.

In conclusion, the thickness of the silver layer has a great influence on the sensor’s performance. The reason is that the core mode of the fiber has a limited penetration depth. When the silver layer thickness increases to a value significantly larger than the penetration depth, the fiber core mode becomes effectively screened from the plasmon and the coupling between the core mode and the plasmon will weaken, resulting in a low coupling efficiency, and as a consequence, low sensitivity, and peak loss. Considering the peak loss amplitude and sensitivity, 40 nm is the optimized silver layer thickness for the sensor.

## 4. Conclusions

In this paper, we have analyzed an exposed-core grapefruit fiber based SPR sensor with a large analyte RI range from 1.33 to 1.42 and different silver layer thicknesses, from 30 to 80 nm, through the finite element method using COMSOL Multiphysics software. Numerical results show that the asymmetrically coated fiber can support two orthogonal resonance peaks (*x*- and *y*-polarized peaks) and the *x*-polarised peak has a higher peak loss and higher sensitivity. Two polarized peaks all shift to the longer wavelength and exhibit increasing peak losses as the analyte RI increasing. The sensor shows a higher sensitivity for the high analyte RI changes than the low and the extremely high wavelength sensitivity 13,500 nm/RIU is obtained. The silver layer thickness has a great influence on the sensor’s performance. When the silver film becomes too thick, the wavelength and amplitude sensitivity all decreased. Thus, when performing experiments, we can tune the peak loss to a desired value by adjusting the silver layer thickness. The designed sensor is based on grapefruit fibers, which have larger air holes than common PCFs and are produced by many manufacturers. It can realize high sensitivity and sensing liquid analytes in the external environment, which is promising to develop a high sensitivity, real-time, fast-response and distributed SPR RI sensor.
